# Deep Learning Accelerators’ Configuration Space Exploration Effect on Performance and Resource Utilization: A Gemmini Case Study

**DOI:** 10.3390/s23052380

**Published:** 2023-02-21

**Authors:** Dennis Agyemanh Nana Gookyi, Eunchong Lee, Kyungho Kim, Sung-Joon Jang, Sang-Seol Lee

**Affiliations:** 1 Electronics Division, Institute for Scientific and Technological Information, Council for Scientific and Industrial Research, Accra, Ghana; 2 Intelligent Image Processing Research Center, Korea Electronics Technology Institute, Seongnam-si 13488, Republic of Korea

**Keywords:** deep learning, hardware accelerators, open-source, Gemmini, systolic array, GEMM, output/weight stationary dataflow, FPGA, image-to-column

## Abstract

Though custom deep learning (DL) hardware accelerators are attractive for making inferences in edge computing devices, their design and implementation remain a challenge. Open-source frameworks exist for exploring DL hardware accelerators. Gemmini is an open-source systolic array generator for agile DL accelerator exploration. This paper details the hardware/software components generated using Gemmini. The general matrix-to-matrix multiplication (GEMM) of different dataflow options, including output/weight stationary (OS/WS), was explored in Gemmini to estimate the performance relative to a CPU implementation. The Gemmini hardware was implemented on an FPGA device to explore the effect of several accelerator parameters, including array size, memory capacity, and the CPU/hardware image-to-column (im2col) module, on metrics such as the area, frequency, and power. This work revealed that regarding the performance, the WS dataflow offered a speedup of 3× relative to the OS dataflow, and the hardware im2col operation offered a speedup of 1.1× relative to the operation on the CPU. For hardware resources, an increase in the array size by a factor of 2 led to an increase in both the area and power by a factor of 3.3, and the im2col module led to an increase in area and power by factors of 1.01 and 1.06, respectively.

## 1. Introduction

The deployment of DL algorithms in applications such as autonomous driving [1], natural language processing [2], robotics [3], and image recognition [4] have been so successful that it has become ubiquitous in the last decade. DL algorithms involve huge amounts of computation to the extent that their implementation in traditional computing devices, such as CPUs, is unfavorable due to the processor’s limitations in power and frequency [5]. The limitations of current CPUs have led to the development of domain-specific architectures (DSAs). DSAs are custom hardware architectures (accelerators) that are designed specifically for a domain of applications [6]. DSAs can achieve higher performance and energy efficiency than CPUs via the ability to customize their control and datapath logic. Investigating DSAs for DL algorithms is an active research and development area for both academia [7,8,9,10] and industry players, including Google [11], Amazon [12], Tesla [13], and Microsoft [14].

There is no denying the fact that DL accelerators have contributed to the efficient implementations of DL algorithms but their design and deployment remain a challenging task. Designing custom DL accelerators for a specific application demands a lot of expertise from the field of hardware/software co-design. Hardware accelerator design and verification at the logic level also take a significant amount of time, which is unacceptable in this era of shorter time-to-market of products [15]. For reasons of difficulty and longer time-to-market of DL accelerators, there is a growing demand for cheaper hardware generators that will make DL accelerator instantiation easier. While there exist several active research programs on the development of DL accelerator generators, the two most relevant ones are the Nvidia Deep Learning Accelerator (NVDLA) [16] and the Gemmini accelerator [17].

The NVDLA is an industry open-source DL accelerator that is configurable and adjustable to many applications based on their resource requirements. NVDLA comes with a hardware/software framework that enables high-level programming of the accelerator and a runtime environment for the actual hardware implementation. Gemmini, on the other hand, is an academic-level, open-source initiative to design and implement a configurable systolic array structure to accelerate the commonly used GEMM operation in DL algorithms.

Though NVDLA is reported to be 3.77× faster at running the ResNet-50 model [18], this study focused on the Gemmini framework for several reasons. The Gemmini framework is better documented than NVDLA, making it easier to implement and understand. It is easier to run simple workloads, such as GEMM, on Gemmini than NVDLA because NVDLA only accepts complete Caffe models. NVDLA only supports a single dataflow, while Gemmini supports a variety of dataflow options, making comparisons possible. Most importantly, Gemmini comes with complete system-level integration of SoC and system software, while NVDLA is a standalone accelerator. System-level integration of DL accelerators is critical for estimating the amount of time needed to move data between the accelerator and the CPU. It also helps with estimating the execution time required for accessing and utilizing shared resources, such as the CPU, accelerator, memory, caches, and the system bus.

This study explored the effects of various configuration parameters of the Gemmini hardware accelerator framework on performance and resource utilization. The contributions of this study include the following:

- Detailing the software and hardware components generated using the Gemmini framework. This was done to make it easier to understand and implement Gemmini since the documentations have a long learning curve.
- The exploration of various GEMM dimensions and dataflow options on different configurations of the Gemmini accelerator is reported to measure performance metrics under various configurations and workloads.
- Mapping the generated register transfer level (RTL) code of various Gemmini configurations on a field programmable gate array (FPGA) device to extract synthesis results, such as area, frequency, and power.
- Demonstrating the effects of various Gemmini configuration options on performance and hardware resource utilization metrics.

The rest of this paper is structured as follows: Section 2 gives some background and related works, Section 3 illustrates the Gemmini framework architecture and ecosystem, Section 4 discusses the methodology involved in exploring the Gemmini architecture, Section 5 elaborates on the performance and hardware resource utilization of several Gemmini hardware configurations, and Section 6 discusses the conclusion and some future research directions.

## 2. Background

This section provides the background on the main building block of DL algorithms, which are the convolution operation, structuring the convolution operation into GEMM, and the method for hardware acceleration of GEMMs known as systolic arrays.

### 2.1. From Convolution to GEMM

**Figure 1:**
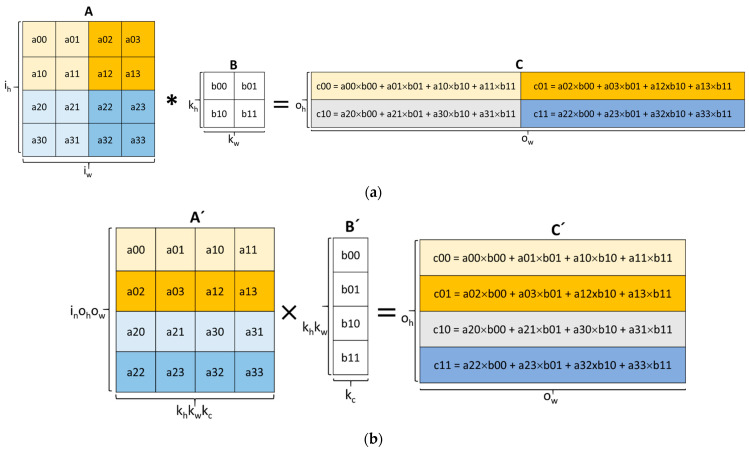

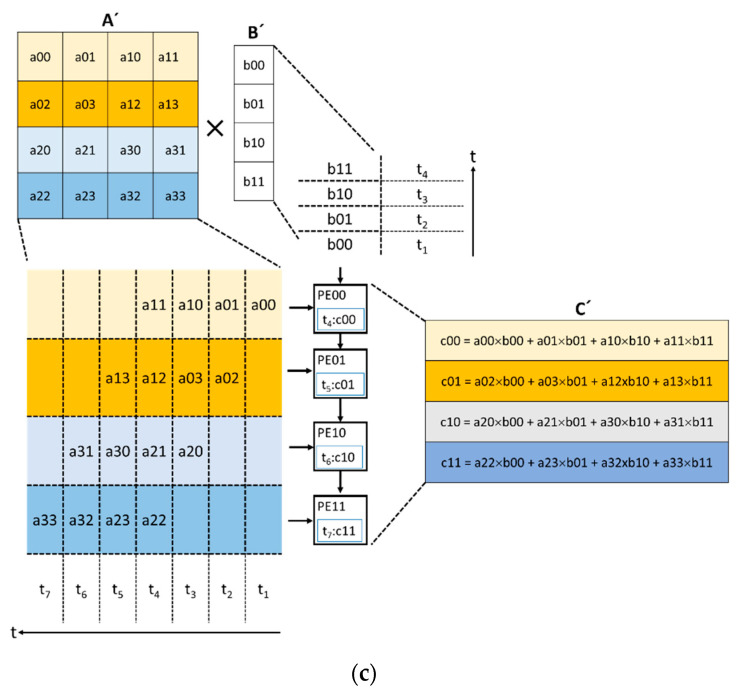
From convolution to systolic array computation: (**a**) direct convolution operation; (**b**) im2col-based GEMM operation; (**c**) example of a systolic array operation.

The convolution operation is one of the main building blocks of DL algorithms and is responsible for feature extraction [19,20]. The convolution operation involves an element-wise dot-product that has an input and a kernel. Figure 1a illustrates an example of a direct convolution between input *A* of *i_h_* height and *i_w_* width and kernel *B* of *k_h_* height and *k_w_* width to produce output *C* of *o_h_* height and *o_w_* width. An output of *C* is produced by the dot-product of *B* and a submatrix of *A* with a height of *k_h_* and width of *k_w_*. The submatrices of *A* are generated by sliding *B* over *A* vertically and horizontally by a parameter known as the stride (*s_h_* for the horizontal stride and *s_w_* for the vertical stride). In the figure, both *s_w_* and *s_h_* have a value of 2. The direct convolution method is very simple and requires less memory but more efficient methods were shown to exist.

A more efficient way of performing convolutions is by lowering the input and kernels into matrices using a process known as im2col and applying the GEMM operation [21,22,23]. Figure 1b shows the transformed input *A* and kernel *B* from Figure 1a into *A*’ and *B*’, respectively, using the im2col method. Each submatrix of *A* with a height of *k_h_* and width of *k_w_* is stretched into a row of *A*’. *A*’ ends up with a dimension of *i_n_o_h_o_w_* × *k_h_k_w_k_c_*, where *i_n_* is the total number of inputs and *k_c_* is the number of kernel channels. The im2col approach is capable of identifying data overlap among the rows of *A*’. The transformed kennel *B*’ is also generated by stretching *B* from Figure 1a into a column. After the im2col transformation, the output *C*’ with a height of *o_h_* and width of *o_w_* is obtained by the matrix multiplication of *A*’ and *B*’. The im2col-GEMM approach can be efficient by using optimized primitives or hardware accelerators.

The im2col-GEMM-based approach to the convolutional operation has led to several innovative methods for hardware acceleration, including the use of a systolic array (SA) [24]. SAs have been around since 1979 but have gained renewed attention since they were used in the Google Tensor Processing Unit (TPU) to accelerate GEMM operations [11]. An SA is made up of a mesh of processing elements (PEs) with an internal multiply–accumulate (MAC) unit. PEs communicate with their neighbors to forward both input data and computed data from the MAC units. In each clock cycle, data flows from the memory to the PEs they are connected to, from PEs to neighboring PEs, and from the last PEs back to the memory. Figure 1c illustrates the operation of a 4 × 1 SA used to multiply the transformed matrices *A*’ and *B*’ to produce *C*’. From the figure, the *A*’ matrix is fed to the SA through the left side while the *B*’ matrix is fed from the top in each clock cycle. Each PE computes the MAC operation until the last input value is received. The final generation of individual elements of matrix *C*’ starts and ends in cycle *t_4_* and cycle *t_7_*, respectively. The SA method implements two main dataflows, which include OS and WS [25]. A dataflow scheme illustrates how the PEs communicate between themselves and how data is accessed and transferred between the memory and the PEs. In the OS dataflow, the results of the partial sums of *C*’ remain in the PEs until the final output is computed. This limits the movement of the partial sums between the PEs and memory. In the WS dataflow, the kernel matrix *B*’ is pre-loaded in the appropriate PEs before the computations start. This enables the reuse of the kernel matrix without accessing memory. The Gemmini accelerator framework, which was the focus of this work, uses the SA method to configure and accelerate GEMM operations of various dimensions.

### 2.2. Related Work

The Gemmini accelerator framework for accelerating DL workloads has been in existence since 2019. The framework is capable of generating an entire system-on-a-chip consisting of processors, memory, peripherals, accelerators, and interconnect fabrics. Since its inception, several researchers have sought to enhance and improve some components of the accelerator. For the SAs of Gemmini to be fully leveraged, the authors in [26] changed the method of computation inside the PEs. They proposed a novel SA architecture, where the carry save adder (CSA) and the carry propagation adder (CPA) are combined to form a hybrid accumulator for the most significant bit (MSB) and the least significant bit (LSB), respectively. On average, their proposal led to improvements of 19.6%, 13.6%, and 16% in the area, delay, and power, respectively, compared with the original implementation of the Gemmini PE computation. To explore the matrix multiplication of different shapes and dimensions, the authors in [27] proposed the configurable matrix multiplication engine for neural network acceleration (CONNA). They effectively implemented a configurable matrix multiplication for different dimensions as opposed to the Gemmini multiplication, which is used for only square matrix dimensions. They implemented CONNA in the Gemmini ecosystems for fair comparisons. Overall, in terms of matrix multiplications, Gemmini recorded 1.56× lower throughput and 3.74× higher power consumption compared with CONNA. 

A few researchers explored the configuration space of the Gemmini accelerator for the performance results of several workloads. The authors of [18] integrated the open-source NVDLA into the Gemmini ecosystem for comparison. They ran the ResNet-50 network workload on similar configurations of NVDLA and Gemmini. They reported that NVDLA was 3.77× faster than Gemmini. The authors of [28] explored the performance of direct convolution on the Gemmini accelerator. They ran various convolution workloads of different dimensions on a single configuration of the Gemmini accelerator and also on a CPU to compare the execution times and energy consumption. They reported that with all the tested convolution dimensions, the Gemmini accelerator was faster and consumed less energy compared with implementations on the CPU.

All related works on Gemmini focus on improving individual components of the accelerator or comparing various workloads on a fixed configuration. There is, therefore, a lack of a thorough configuration space exploration of the accelerator on workloads involving the basic building block of DL algorithms, which is the GEMM operation. Moreover, the effects of different configurations of the accelerator on hardware resource consumption are not usually reported. To date, the only hardware resource utilization of the Gemmini accelerator can be found in the original papers [29]. The original papers reported application-specific integrated circuit (ASIC) results in terms of the area and power by varying a single design parameter, including the SA dimension and dataflow. The studies investigated only two SA dimensions, which included the baseline 16 × 16 and 32 × 32. The original studies also ignored FPGA results, which have become integral in prototyping DL algorithms due to their flexibility and shorter time to market. The original studies also investigated the performance of various DNNs, including Resnet-152, Resnet-50, and MobileNet, on a single configuration of the Gemmini accelerator. However, the studies neglected the performance of GEMM operations, which is the basic block mapped to the systolic array of the Gemmini accelerator. Though the GEMM operation is simple compared with an entire neural network, it is essential to investigate its performance in order to be able to improve on the accelerator, specifically the systolic array. This study investigated the performance metrics of different GEMM matrix sizes on various configurations of the Gemmini accelerator. This was done to observe the effects of the Gemmini configuration parameters, such as SA sizes, dataflow options, and im2col operation, on performance regarding various GEMM workloads. This study also aimed to serve as a milestone regarding mapping various Gemmini configurations on a commercial FPGA device to report their effects on hardware resources, such as area, frequency, and power consumption. This study also explored the performance–resource utilization metrics of the various configurations of the Gemmini accelerator.

## 3. The Gemmini Hardware Architecture Template

Gemmini is part of the open-source RISC-V Chipyard framework [30] that is continuously under development at the University of California, Berkeley. The RISC-V chipyard framework is the best option for custom hardware architecture design because it contains several open-source tools and libraries for SoC design. The framework mainly uses Chisel [31], which is a new hardware description language (HDL) that is designed to implement configurable hardware designs using parameters. The Chisel language is embedded in Scala to support functional primitives and high-level object-oriented programming. The Gemmini framework is a flexible systolic array accelerator generator that supports several configuration options and targets ASIC implementations. It not only generates systolic arrays but also an entire SoC for simulations and implementations. The Gemmini framework customizes the SoC based on parameters such as the host processor, bus width, memory capacity, number of memory banks, pipeline depth, data bit width, systolic array dimension, and data flow.

This section documents the detailed hardware architectures of the modules generated by the Gemmini framework. Gemmini is described in the Chisel HDL, which can be compiled to generate Verilog HDL codes. Since it is difficult to understand the generated Verilog codes, this section describes some of the modules in the Gemmini hardware architecture.

**Figure 2:**
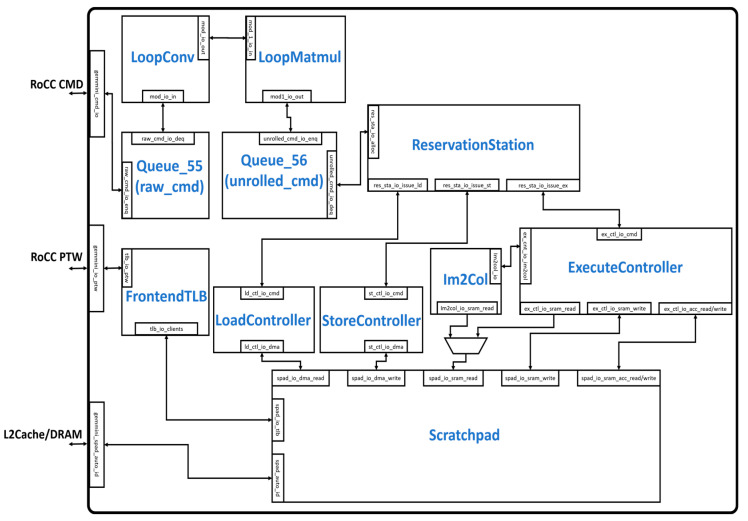
Modules in the Gemmini hardware architecture.

Figure 2 illustrates the overall hardware architecture of the Gemmini accelerator. The accelerator communicates with the outside world through the Rocket Custom Coprocessor (RoCC) interface and the cache/DRAM interface. The RoCC interface sends instructions to the accelerator in the form of RoCC commands (CMD), which are buffered by the Queue_55 module. The CMD is then unrolled into various tasks by the LoopConv and LoopMatmul modules before being buffered by the Queue_56 module and subsequently sent to the ReservationStation module. The ReservationStation module reorders commands into a series of load, store, and execute tasks and distributes them to the LoadController, StoreController, and ExecuteController respectively. The LoadController and the StoreController interact with the DMA read and DMA write interface of the Scratchpad module, respectively. The ExecuteController module interacts with the SRAM read/write and the accumulator read/write interface of the Scratchpad module. Depending on the configuration, the im2col module also interacts with the SRAM read interface of the Scratchpad module. The Scratchpad module interacts with the cache/DRAM interface to transfer data according to the addresses generated by the FrontendTLB module.

### 3.1. The RoCC Interface

**Figure 3:**
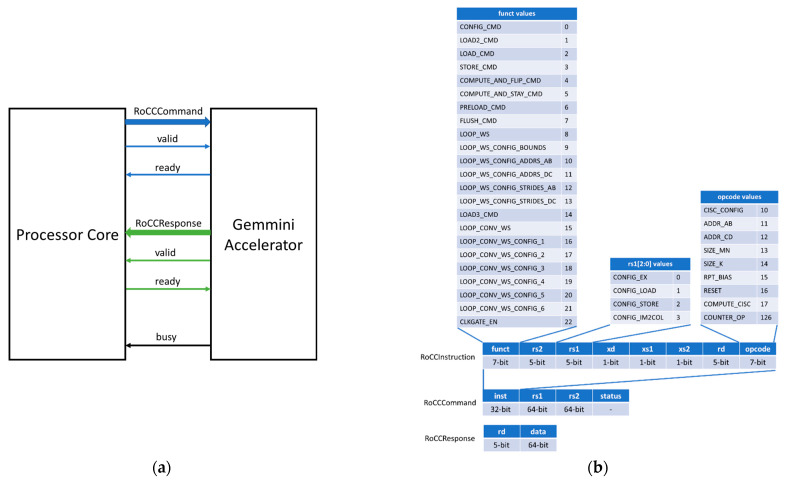
The RoCC interface: (**a**) the RoCC interface signals; (**b**) the RoCC command and response formats.

The RoCC interface is designed to allow for decoupled (read/valid) communication between the Gemmini accelerator and the processor core. The processor core can be configured to be the RISC-V Berkeley Out-of-Order Machine (BOOM) [32] or the RISC-V Rocket core [33]. As illustrated in Figure 3a, the basic structure of the RoCC interface consists of the command (RoCCCommand) and response (RoCCResponse) signals. The RISC-V processor core sends commands and related data to the Gemmini accelerator through the RoCCCommand interface while it receives results from the accelerator through the RoCCResponse interface. The format of the RoCCCommand and RoCCResponse is shown in Figure 3b.

The RoCCCommand is divided into a 32-bit instruction field (inst) together with two 64-bit registers rs1 and rs2. The inst (RoCCInstruction) field is divided into the function (funct7), operation code (opcode), source registers (rs1 and rs2), destination register (rd), and control signals (xd, xs1, and xs2). The control signals ensure the correct use of the RoCC interface and serve as valid signals for source and destination registers.

The RoCCResponse interface is divided into the rd and data fields. The rd field specifies the destination register of the response and the data field specifies the content to be written to the rd register.

### 3.2. LoopConv and LoopMatmul Modules

**Figure 4:**
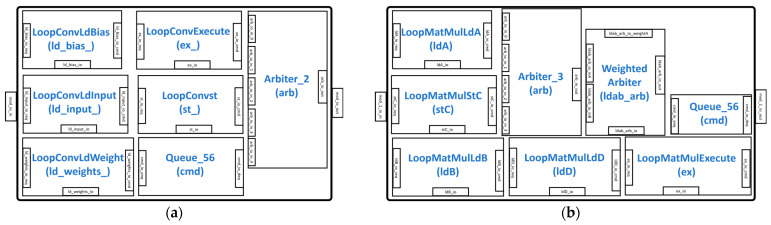
Command unrolling modules: (**a**) LoopConv module; (**b**) LoopMatmul module.

The LoopConv and LoopMatmul modules are responsible for unrolling large convolutions and matrix multiplications (matmuls), respectively, to fit the systolic dimension of DIM × DIM. The LoopConv shown in Figure 4a consists of modules, such as LoopConvLdBias, LoopConvLdWeight, and LoopConvLdInput, for signifying instructions for the convolution operation bias, weight, and input, respectively. The LoopConvExecute and LoopConvst modules are instructions for executing the convolution operation and storing results respectively. In the LoopMalmul module shown in Figure 4b, the LoopMatmulLdA, LoopMatmulLdB, and LoopMatmulLdD specify the loading of matrices *A*, *B*, and *D*, respectively, while the LoopMatmulExecute module generates commands for the matrix multiplication. The LoopMatmulStC generates commands for storing the results of matrix *C*. The Queue_56 module in both LoopConv and LoopMatmul is used to buffer the input command signals. The unrolled commands from the LoopConv and LoopMatmul modules are assigned to the ReservationStation to be distributed to the controllers.

### 3.3. LoadController and StoreController Modules

**Figure 5:**
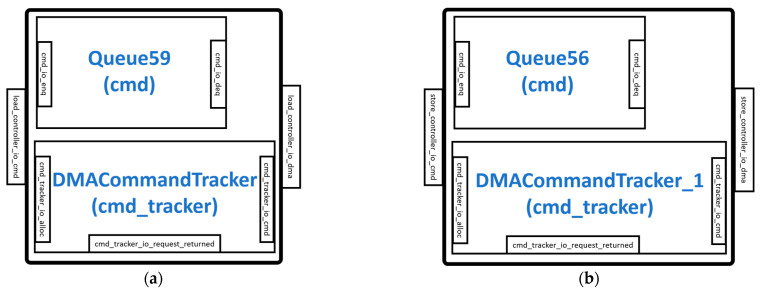
DMA controllers: (**a**) LoadController module; (**b**) StoreController module.

The LoadController and StoreController are similar in design and are responsible for the instructions that move data from the DRAM to the Gemmini scratchpad and from the scratchpad to the DRAM, respectively. The LoadController shown in Figure 5a connects to the scratchpad DMA read interface while the StoreController shown in Figure 5b connects to the scratchpad DMA write interface. Both controllers use a finite state machine (FSM) with three states, namely, *waiting_for_cmd*, *sending_rows*, and *waiting_for_dma_req_ready* states, to control their running modes. In the *waiting_for_cmd* state, the controllers configure themselves and load the commands from the ReservationStation module into the DMACommandTracker and DMACommandTracker_1 for the LoadController and StoreController, respectively. The DMACommandTracker and the DMACommandTracker_1 keep track of the total amount of data to be loaded into the scratchpad and stored in the DRAM, respectively. In the *waiting_for_req_ready* state, the controllers suspend until the DMA configures itself to read and write data. In the *sending_row* state, the controllers keep working until the right amount of data is loaded or stored according to the values in the DMA command trackers.

### 3.4. im2col and ExecuteController Modules

The im2col and the ExecuteController modules connect to the SRAMs inside the scratchpad module. The im2col module is optional and only generated in the hardware if configured to do so; otherwise, the im2col operation is handled by the processor core.

**Figure 6:**
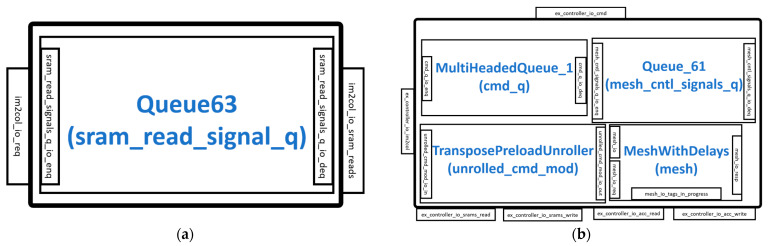
SRAM controllers: (**a**) im2col module; (**b**) ExecuteController module.

When the Gemmini accelerator is configured to have an im2col module, the interface connects and receives instructions from the ExecuteController module and also reads data from the SRAM read interface of the scratchpad module, as shown in Figure 6a. The read data is then fed to the ExecuteController module. The im2col module is an address generator that uses an FSM to make sure that the PEs inside the systolic array of the ExecuteController are used as much as possible. The states in the im2col FSM include *nothing_to_do*, *waiting_for_im2col*, *preparing_im2col*, and *im2col_done*.

The various modules and interfaces of the ExecuteController architecture are illustrated in Figure 6b. The controller connects to the im2col interface and the SRAM interfaces of the scratchpad. The execution command from the ReservationStation is unrolled by the TransposePreloadUnroller module and stored in the MultiHeadedQueue_1 module. The MultiHeadedQueue_1 module is a buffer that separates the instructions into commands for the Scratchpad module and the MeshWithDelays module. The instructions or commands for the scratchpad module are sent to extract data from the SRAMs. The commands for the MeshWithDelays are first buffered in the Queue_61 module before being sent to control the systolic array and other components in the module.

**Figure 7:**
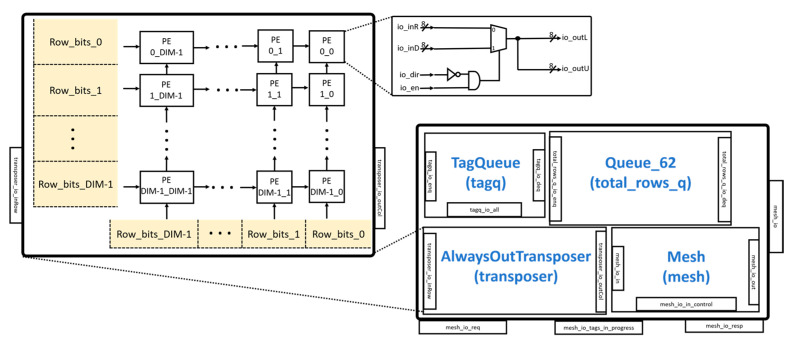
MeshWithDelays module.

The MeshWithDelays module of the ExecuteController is illustrated in Figure 7. It consists of modules such as the Mesh and AlwaysOutTransposer.

The AlwaysOutTransposer is used to transpose a matrix before it enters the MeshWithDelays module. The AlwaysOutTransposer is implemented as a systolic array with configurable DIM × DIM PEs, as shown in Figure 7. The module receives input from both the left side and the bottom of the systolic array. The systolic array transfers the inputs from the left side to the right side using a total of DIM cycles. The systolic array then transfers the inputs from the bottom to the top using a total of DIM cycles. Each PE uses a multiplexer to select the left or bottom inputs.

**Figure 8:**
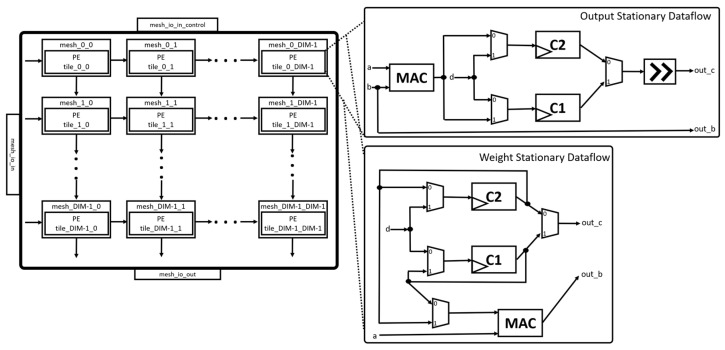
Mesh module.

The Mesh module is shown in Figure 8. The module implements the systolic array of the Gemmini accelerator with configurable mesh rows and mesh columns. There are delay registers at each mesh output to enable each input from one matrix to arrive at the same time as the input of another matrix before multiplication. In the Mesh module, there is a hierarchy in which each mesh contains a tile that is made up of PEs. The PEs are the main computational unit that implements a combinational logic for the MAC unit. The PEs can also be configured to employ different dataflows, such as OS, WS, or both, as shown in the figure. The Mesh module is designed to perform matmul operations of DIM × DIM size. The DIM parameter is the number of PEs in the width of the systolic array and has a default value of 16. The Mesh module takes inputs from the AlwaysOutTransposer unit with the output fed to the SRAM/accumulator of the Scratchpad module. The Mesh module can be configured to have several pipeline stages.

### 3.5. Scratchpad Module

**Figure 9:**
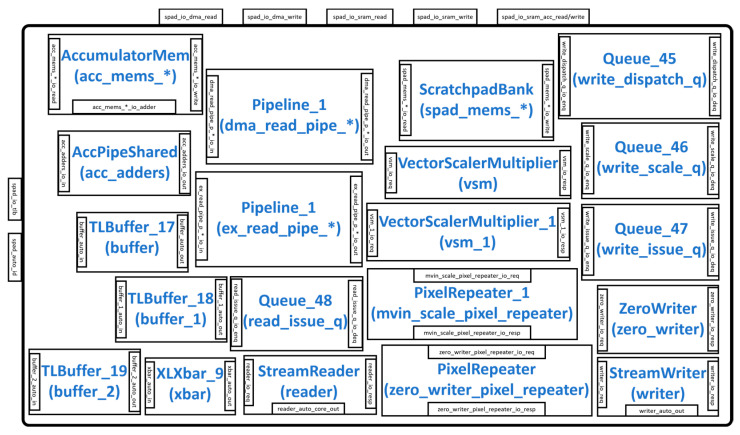
Scratchpad module.

The scratchpad module shown in Figure 9 is responsible for storing data that is generated during the matrix multiplication or convolution operation. It consists of input/output (IO) interfaces, including spad_io_tlb (connects to the FrontTLB module), spad_auto_id (connects to the L2cache/DRAM), spad_io_dma_read/write (connects to the DMA), spad_io_sram_read/write (connects to the ScratchpadBank), and spad_io_acc_read/write (connects to the AccumulatorMem). Several pipeline modules (Pipeline_1) can be configured to pipeline the DMA and the ExecuteController read operations. The ScratchpadBank and AccumulatorMem send their data to the DRAM by first writing it into the StreamWriter module. The DMA control signals interact with the StreamReader and StreamWriter modules to read or write their content according to the addresses in the FrontTLB, which is buffered. When configured to perform vector multiplications, the data in the StreamReader is sent to the VectorScalerMultiplier/VectorScalerultiplier_1 for computation, and the results are stored in the AccumulatorMem or ScratchpadBank. The ZeroWriter module implements a function that writes 0 to all specified memory locations.

**Figure 10:**
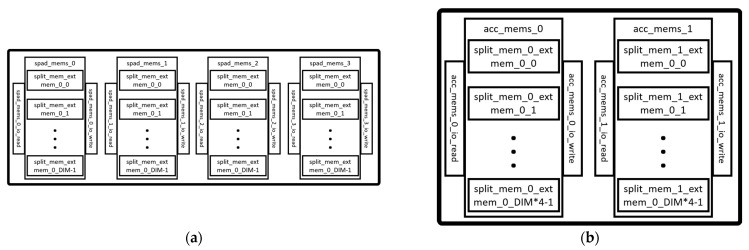
Scratchpad memories: (**a**) ScratchpadBank module; (**b**) AccumulatorMem module.

Figure 10a shows the arrangement of the ScratchpadBank in the Scratchpad module, which is made up of simple SRAMs. The number of banks is configurable but usually consists of four banks for storing matrices *A*, *B*, *D*, and *C*. Each bank has a default data width of 8 and a depth of DIM.

Figure 10b shows the arrangement of the AccumulatorMem, which is also made up of simple SRAMs. The module is also configurable but the default consists of two accumulators for storing the intermediate or final results of the matrix multiplication or convolution operation. The data width of each accumulator is a default value of 32, while the depth is DIM × 4. In addition to the SRAMs, the accumulators have a set of adders located in the AccPipeShared of the scratchpad module. They also have an internal scaler for transforming the 32-bit data width into an 8-bit data width.

## 4. Methodology

**Figure 11:**
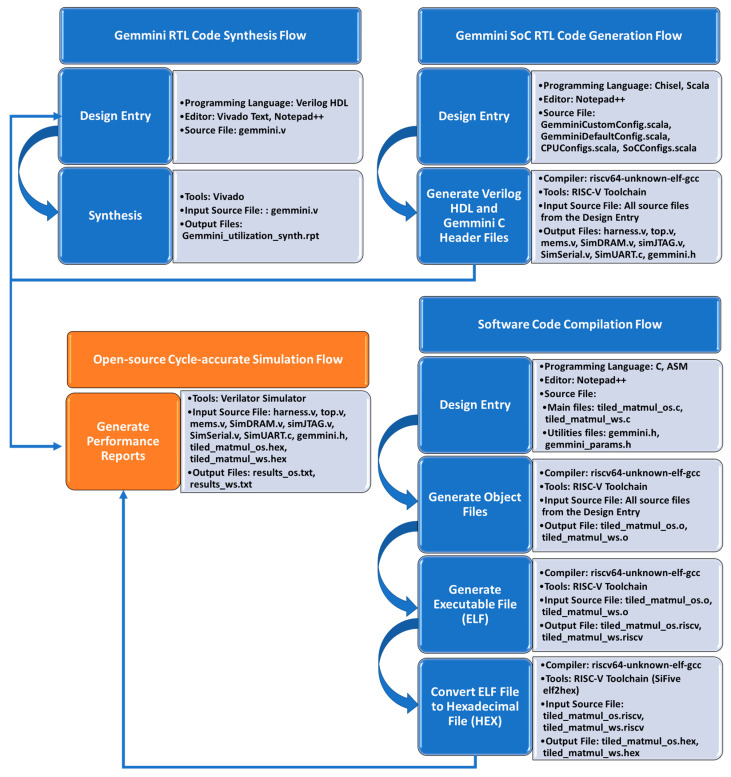
Workflow for exploring Gemmini accelerators.

This work explored the effects of several configuration parameters of the Gemmini accelerator on both the performance and hardware resource utilization by using a development framework that was made up of a combination of both open-source and proprietary tools. The development framework flow for this work is illustrated in Figure 11. The flow is organized into three interconnected categories, which include the Gemmini SoC RTL code Generation Flow, the Software Code Compilation Flow, and the Gemmini RTL Code Synthesis Flow. This section details the workflows giving the steps involved and the tools used in each step.

### 4.1. Gemmini SoC RTL Code Generation Flow

**Figure 12:**
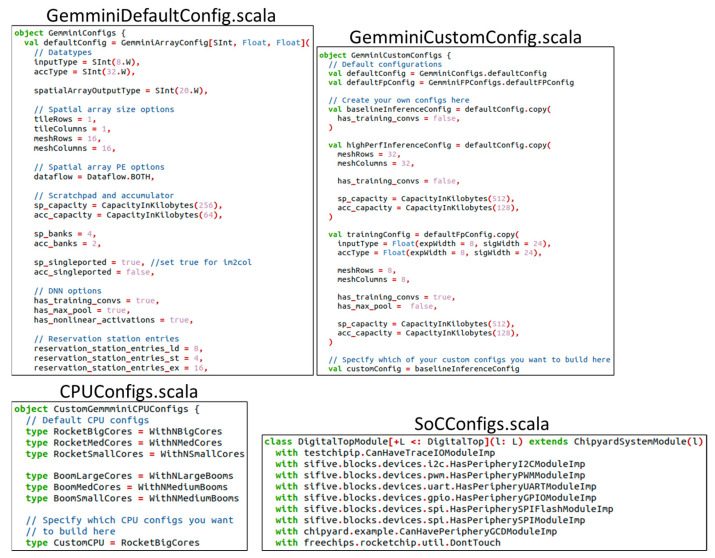
Gemmini SoC generation configuration files.

Information on the generation of the Gemmini SoC RTL code can be found in the Gemmini repository [34]. The repository also contains information on setting up and installing the RISC-V toolchain for the SoC generation. The Gemmini SoC can be configured by editing Scala files that define modules of the SoC. Sections of the Scala files are shown in Figure 12. These files include the GemminiDefaultConfig.scala for customizing the Gemmini accelerator, GemminiCustomConfig.scala for choosing the custom configuration to build, CPUConfigs.scala for selecting the type of RISC-V CPU, and SoCConfigs.scala for selecting some peripherals. Running the configuration commands generates the Gemmini SoC Verilog codes together with a header file (gemmini.h) made up of customized C code Gemmini functions.

**Figure 13:**
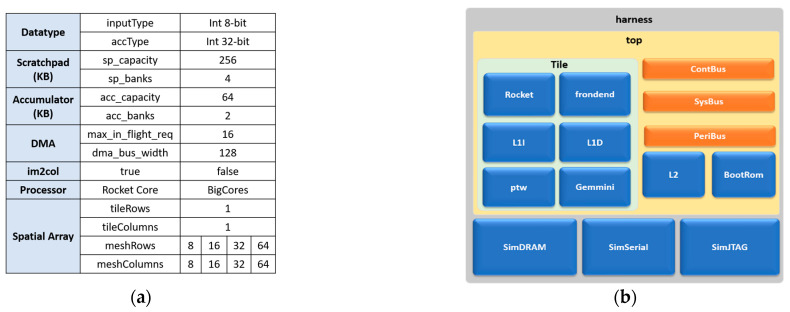
Gemmini SoC generation: (**a**) Gemmini configuration parameters; (**b**) Gemmini SoC generated modules.

Figure 13a illustrates the configuration parameters selected for generating the Gemmini accelerators for this work. The processor selected was the in-order five-stage Rocket core, which is integrated with the accelerator. The Gemmini accelerator was composed of a systolic array with one tile and a variety of meshes, including 8 × 8, 16 × 16, 32 × 32, and 64 × 64. The scratchpad was made up of four banks each with 256 KB memory, while the accumulator was made up of two banks, each with 64 KB memory. Each systolic array was generated with and without a hardware im2col module. The overall architecture for simulating the Gemmini SoC is shown in Figure 13b, which consisted of a tile made up of the Rocket core and the Gemmini accelerator with simulation modules for DRAM, serial communication, UART communication, and JTAG. All the modules generated from this step were fed to an open-source cycle-accurate simulator, while only the Gemmini module was fed to a proprietary/commercial synthesis tool.

### 4.2. Software Code Compilation Flow

The Gemmini hardware accelerator can be controlled in several ways. The control or programming of the Gemmini accelerator can be achieved at three different levels, including high-level programming of compiling ONNX [35] models, mid-level programming using kernel libraries, and low-level programming using assembly or direct machine configuration. To investigate the performance of GEMM on various configurations of the Gemmini accelerator, this work selected the mid-level programming of the accelerator.

**Figure 14:**
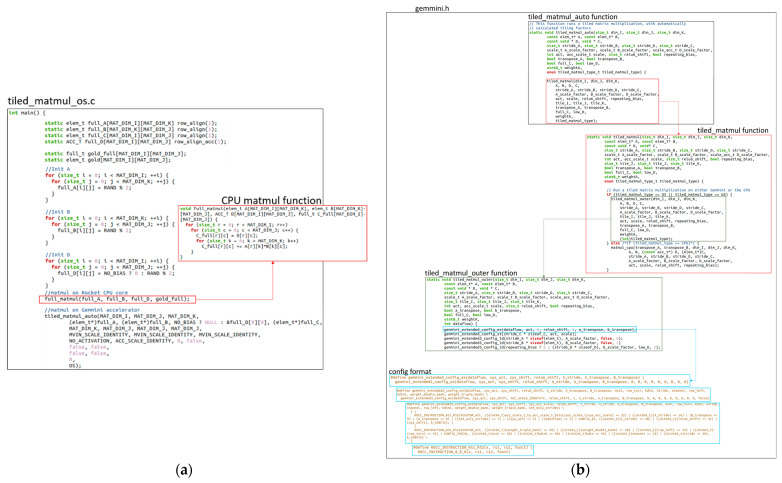
Gemmini software code file: (**a**) program source file; (**b**) Gemmini header file.

This work compiled bare-metal matrix multiplication programs that could be executed in the Gemmini hardware or the Rocket CPU core. The programs included the tiled_matmul_os.c for the OS dataflow option and tiled_matmul_ws.c for the WS dataflow option. A section of the tiled_matmul_os.c file is shown in Figure 14a. For each of the programs, the input matrix dimension was varied (2 × 2, 8 × 8, 16 × 16, 32 × 32, and 128 × 128) and runs were executed on both the Rocket CPU core and Gemmini accelerators with systolic array configurations of 8 × 8, 16 × 16, 32 × 32, and 64 × 64. The program started by initializing the matrices with random values before calling the full_matmul function for the execution of matrix multiplication on the Rocket CPU. The program then called the tiled_matmul_auto function for the execution of matrix multiplication on the Gemmini accelerator. The tiled_matmul_auto function was located in the gemmini.h header file, which was generated together with the Gemmini SoC Verilog files according to the steps in Section 4.1. Some sections of the gemmini.h header file are illustrated in Figure 14b. The tiled_matmul_auto function ran matrix multiplication of dimensions that were higher than the systolic array by automatically calculating the tiling factors. The function then called the tiled_matmul function with hardcoded tiling parameters, which subsequently called the tiled_matmul_outer function. The tiled_matmul_outer made a series of configuration function calls, including gemmini_extended3_config_ld (for moving matrix *A*, *B*, and *D* from DRAM to the Scratchpad), gemmini_extended_config_st (for moving data from the Scratchpad to the DRAM), and gemmini_extended_config_ex (for executing the matrix multiplication in the systolic array of the ExecuteController module). Each of the configuration functions was arranged into the RoCCommand format (Figure 3) and was transferred to the Gemmini accelerator through the RoCC interface.

The RISC-V compiler (riscv64-unknown-elf-gcc version) was used to compile the tile_matmul_os.c and tiled_matmul_ws.c programs together with a linker file that pointed to the base address of the instruction memory. Executable (.exe) files were generated and converted to hexadecimal files (.hex) tiled_matmul_os.hex and tiled_matmul_ws.hex using the SiFive elf2hex program [36].

The tiled_matmul_os.hex and tiled_matmul_ws.hex files together with the output Verilog files generated according to Section 4.1 were fed as inputs to the open-source Verilator tool [37] for the simulation. The Verilator simulator loaded the hexadecimal files into the designated location of the DRAM simulation module, which prompted the Rocket CPU to begin executing instructions starting from the base address. The simulator reported the performance of executing the matrix operations on both the Rocket CPU core and the Gemmini accelerators for comparison.

### 4.3. Gemmini RTL Code Synthesis Flow

The Verilog files of the Gemmini accelerators were synthesized using the commercial tool Vivado v2020.2, which is a hardware computer-aided design (CAD) software that converts HDLs into gate-level netlist files. Four Gemmini accelerators were generated based on various systolic array sizes, namely, 8 × 8, 16 × 16, 32 × 32, and 64 × 64, for comparison. These codes generated according to Section 4.1 were fed as inputs to the synthesis software. Each of the accelerators had an option for including an im2col module or not. This work synthesized each of the Gemmini accelerators using the Virtex UltraScale+ XCVU19P-FSVA3824-1-E FPGA device. The synthesis report of each Gemmini accelerator produced the resources occupied, the maximum frequency, and the power consumption.

## 5. Experimental Results

This section documents the experimental results obtained by following the methodology of Section 4 to estimate various Gemmini configuration effects on the performance and hardware resource utilization. This section extends results from previous work [38]. A GitHub repository [39] is available with codes, documents, and instructions for reproducing the results of this work.

### 5.1. Gemmini Performance Analysis

The GEMM operations of different sizes, which represent the basic operations of DL algorithms, were used as evaluation workloads to evaluate the performance of various Gemmini accelerators. The GEMM workloads were made up of different matrix dimensions, namely, 2 × 2, 8 × 8, 16 × 16, 32 × 32, and 128 × 128. These GEMM workload programs were run on various Gemmini accelerators with different systolic array dimensions, namely, 8 × 8, 16 × 16, 32 × 32, and 64 × 64. Each of the accelerators had the option to perform the im2col operation in either the CPU core or the Gemmini hardware.

The Rocket core, which implements the standard RISC-V Instruction Set Architecture (ISA), including integer, multiplication, and compressed extension, was chosen as the baseline CPU for comparison. The GEMM workloads were therefore run on both the Rocket core and the Gemmini accelerators at an operation frequency of 100 MHz on the Verilator simulator. The Verilator simulator reported the total execution times of the various GEMM workloads on the Rocket core and the Gemmini accelerators

**Figure 15:**
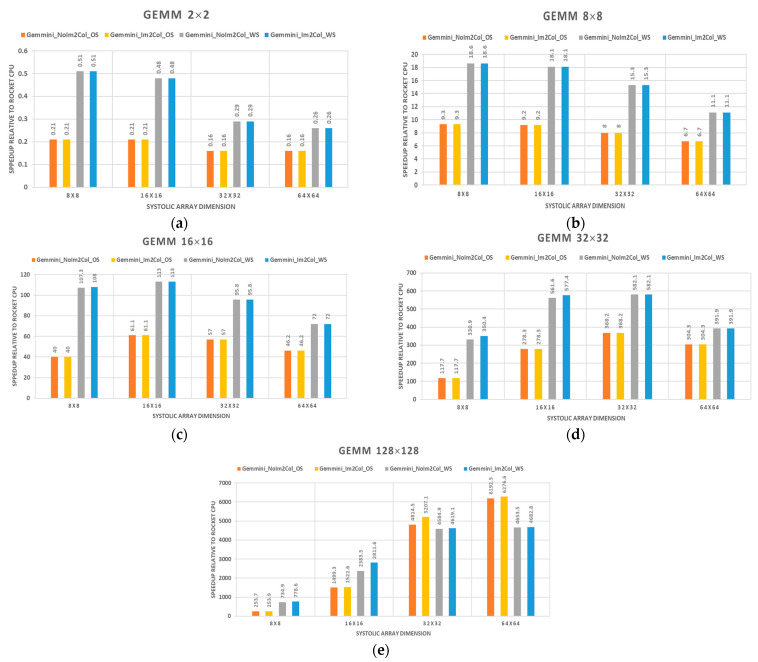
Gemmini accelerator speedups relative to the baseline Rocket CPU core (above 1 indicates a speedup while less than 1 indicates a slowdown): (**a**) 2 × 2 GEMM; (**b**) 8 × 8 GEMM; (**c**) 16 × 16 GEMM; (**d**) 32 × 32 GEMM; (**e**) 128 × 128 GEMM.

Table 1 illustrates the performances in terms of the execution time of various GEMM sizes on Gemmini accelerators with different systolic array dimensions and the baseline Rocket CPU core. Figure 15 extends Table 1 by illustrating the speedups of Gemmini accelerators with different systolic array dimensions in relation to the baseline Rocket CPU core. The speedup is the ratio of the execution time of the baseline Rocket CPU to the Gemmini accelerator. From the figure, values above 1 indicate a speedup while values below 1 indicate a slowdown relative to the Rocket CPU core.

Figure 15a shows the speedup of the GEMM operation with a 2 × 2 matrix dimension workload on the various Gemmini accelerator relative to the Rocket core. The Gemmini_No_Im2Col_OS and Gemmini_Im2Col_OS labels represent OS dataflow configurations with an im2col operation in the CPU and the Gemmini hardware, respectively. Furthermore, the Gemmini_No_Im2Col_WS and Gemmini_Im2Col_WS labels represent WS dataflow configurations with an im2col operation in the CPU and the Gemmini hardware, respectively. From the figure, it can be observed that the Rocket core outperformed all the Gemmini accelerators. There was a slowdown in all the Gemmini accelerators because the matrix size of 2 × 2 was less than the systolic array dimensions of 8 × 8, 16 × 16, 32 × 32, and 64 × 64. The Gemmini accelerator architecture was designed for inputs to be pushed through the array at each clock cycle depending on the dimension of the systolic array. With a matrix size less than the systolic array dimension, the final output result was calculated by pushing the matrix inputs to the last PE of the systolic array. With the 2 × 2 matrix dimension, the GEMM operation was faster using the Rocket CPU instructions than on the Gemmini accelerators. The worst performing accelerator was the 64 × 64 systolic array configuration with an OS dataflow that performs 6× below that of the baseline Rocket core.

Figure 15b–e illustrate the performances of the GEMM workload with matrix sizes of 8 × 8, 16 × 16, 32 × 32, and 128 × 128 on Gemmini accelerators with systolic array dimensions of 8 × 8, 16 × 16, 32 × 32, and 64 × 64, respectively. From the figures, all the Gemmini accelerators reported speedups above 1, which indicated that they outperformed the baseline Rocket CPU core.

It can be seen that for each GEMM operation, the WS dataflow option offered a significant speedup compared with the OS dataflow of an average of 2×, except for the 128 × 128 matrix operation on 32 × 32 and 64 × 64 systolic array dimensions. This was because the WS dataflow option preloaded one of the matrices and avoided expensive memory accesses, thereby reducing the execution time. However, with larger matrix dimensions (128 × 128), the OS dataflow performed complete computations in the PEs, which is faster than the WS dataflow option of partial computations.

It can also be observed that doubling the GEMM matrix size resulted in an average speedup of 2× relative to the previous GEMM workload. Again, for each GEMM workload, the highest speedup relative to the Rocket CPU core was reported by a Gemmini configuration having the same systolic array dimension as the GEMM matrix size. For example, in the 32 × 32 GEMM operation, the Gemmini 32 × 32 systolic array configuration with OS dataflow was 3.1×, 1.3×, and 1.2× faster than the 8 × 8, 16 × 16, and 64 × 64 systolic arrays, respectively. Lastly, the im2col operation in the Gemmini hardware was only significant with very high matrix sizes, as observed in the 128 × 128 GEMM operation of Figure 15e. From the figure, the average speedup offered by the hardware im2col module was 1.1× relative to delegating the operation to the CPU of the SoC.

Generally, the performance of the various systolic arrays can be attributed to the external memory bandwidth. Ideally, the input bandwidth should increase with the side length of the systolic array. Moreover, the latency to push data through the systolic array increases with an increase in the array dimension. For that matter, the performance efficiency is highest when the external memory bandwidth matches the systolic array dimension. For instance, given a 64 × 64 array dimension, the input bandwidth is twice that of a 32 × 32 array dimension. Given a 64 × 64 GEMM workload on a 64 × 64 systolic array architecture, ideally, it takes 64 clock cycles for the elements of the first row of inputs to traverse the array and another 64 clock cycles for the partial sums to travel down the array and accumulate, resulting in a total of 128 clock cycles. On the other hand, a 64 × 64 GEMM workload on a 32 × 32 systolic array architecture will ideally require about 256 clock cycles, excluding memory access latency. This means that the 64 × 64 array is twice as fast as the 32 × 32 array. However, a 32 × 32 GEMM workload on a 64 × 64 systolic array architecture is twice as slow as a 32 × 32 systolic array architecture. This is because even though the bandwidth of the 32 × 32 GEMM workload is half that of the 64 × 64 systolic array, the input data still have to traverse all the way to the end of the array, increasing the latency and therefore making the 64 × 64 systolic array less effective than the 32 × 32 systolic array. This explanation is given to draw attention to the fact that the systolic array dimension is as effective as the memory bandwidth it is connected to. If the bandwidth is less than the systolic array dimension, the hardware accelerator will be deemed inefficient, as most of the processing elements will be relegated to the task of forwarding data rather than computation.

Overall, some important lessons from conducting the experiment include the following: for the Gemmini accelerator to be effective, the GEMM sizes should be above the systolic array dimension (most effective when they are equal); use WS dataflow for smaller GEMM sizes and the OS dataflow for larger GEMM sizes; only include the Gemmini hardware im2col module for very large GEMM sizes; and lastly, the accelerator should not be used for GEMM dimensions below 2 × 2.

### 5.2. Gemmini Hardware Resource Utilization Analysis

To investigate various Gemmini configuration options for hardware resource utilization, the accelerators were synthesized using the Vivado 2020.2 software with the Virtex UltraScale+ XCVU19P-FSVA3824-1-E FPGA device, as described in Section 4.3. The FPGA device selected was high-end with the following resources: 4085760 configurable logic block look-up tables (CLB LUTs), 8171520 CLB registers, 2160 block RAM (BRAM) tiles, and 3840 digital signal processors (DSPs). This experiment explored the effects of different systolic array dimensions on the resource consumption. A total of four systolic array dimensions, namely, 8 × 8, 16 × 16, 32 × 32, and 64 × 64, each with and without a hardware im2col module, were synthesized to extract the hardware resources, maximum frequency, and power consumption. Table 2 shows the results of synthesizing the various Gemmini configurations. The 8×8_CPU_im2col and 8×8_Gemmini_im2col labels indicate the systolic array dimension was 8 × 8 with the im2col operation delegated to either the CPU or the Gemmini hardware, respectively. With all the Gemmini accelerators, the default Vivado synthesis options were maintained with no specific optimization mappings to the FPGA fabric.

The CLBs of an FPGA are responsible for its programmability. The XCVU19P-FSVA3824-1-E CLB was organized into a slice, which was made up of either six-inputs-to-one-output or five-inputs-to-two-outputs LUTs together with a flip-flop (FF), adders, and registers. From Table 2, the accelerator with an SA dimension of 8 × 8 consumed the fewest LUTs and registers, with utilizations of less than 2.3% and 0.5%, respectively, while the 64 × 64 dimension used the most LUTs and registers, with utilizations above 71% and 7%, respectively, which makes it impractical for low-cost devices. It can be observed that doubling the SA dimension led to an increase in the LUTs and registers by average factors of 3.3 and 2.7, respectively. The im2col module in hardware increased the LUTs by a factor of 1.01, while it led to a negligible reduction in the number of registers.

The MAC operation in the SAs makes use of the multiplication operator, which is very expensive to implement with LUTs and registers. For that matter, the FPGA device comes with customized DSP blocks for multiplication and accumulation. The DSP block was designed to improve the efficiency and speed of functions. From the table, the 8 × 8 SA configuration used the fewest DSP blocks, with a utilization of less than 4%, while the 64 × 64 SA configuration used the highest number of DSP blocks, with a utilization above 6%. Doubling the SA dimension led to an increase in the DSP blocks by an average factor of 1.2. It can be observed that the hardware im2col module did not affect the DSP blocks because it is a simple address generator made up of just adders and registers.

The Vivado synthesis tool implements memory elements using either distributed RAM or BRAM. Distributed RAMs are often implemented with the CLB LUTs and, therefore, were not independently reported. The BRAM of the FPGA device could store a maximum of 36 Kb of data and can be configured as one 36 Kb RAM (RAMB36K) or two 18 Kb RAMs (RAMB18K). Each BRAM tile, therefore, indicated one RAMB36K or two RAMB18K. The BRAM tiles were mostly used by the ScratchpadBank and the AccumulatorMem modules of the Gemmini accelerator. From Figure 10, the ScratchpadBank was made up of four banks, with each bank consisting of DIM rows, while the AccumulatorMem was made up of two banks, with each bank consisting of DIM × 4 rows. Ideally, to implement all the memory elements in BRAM tiles, the SA configurations of 8 × 8, 16 × 16, 32 × 32, and 64 × 64 should use 96, 160, 288, and 544 tiles, respectively. Since no optimization options were selected during the synthesis, the Vivado tool implemented each row of the ScratchpadBank and the AccumulatorMem in one BRAM tile or distributed BRAM. From Table 2, the SA 8 × 8 used 96 tiles (96 RAMB36K for both ScratchpadBank and AccumulatorMem), SA 16 × 16 used 128 tiles (64 RAMB36K for ScratchpadBank and 128 RAMB18K for AccumulatorMem), SA 32 × 32 used 64 tiles (128 RAMB18K for ScratchpadBank, while the AccumulatorMem was implemented with distributed RAM), and SA 64 × 64 used 128 tiles (256 RAMB18K for ScratchpadBank, while the AccumulatorMem was implemented with distributed RAM). The hardware im2col module did not affect the number of BRAM tiles.

Estimating the maximum frequency of design using the Vivado tool is not as straightforward as in the Xilinx Integrated Synthesis Environment (ISE), which was discontinued. To use the Vivado tool to estimate the maximum frequency, a timing constraint file with an estimate for the frequency was provided before running the tool and checking for both the setup and hold time violations. The estimated frequency was increased anytime a timing violation was reported. This was the approach used in this work to obtain the maximum frequency of each Gemmini configuration. From Table 2, the 64 × 64 SA dimension accelerator achieved the highest frequency, with a critical path delay of 18 ns, which translated to 55.6 MHz.

The Vivado tool reports both the static power and the dynamic power used by a design. Static power is consumed when a circuit is non-functional (in a steady state), while dynamic power is consumed when the circuit is operational and switching between states. The dynamic power of a circuit is proportional to the operational frequency used by the circuit. This work maintained all the default settings of the Vivado tool for power analysis of the Gemmini configurations and reported only the dynamic power consumption since the static power was constant given the use of the same default settings. For a fair comparison, the operational frequency for the power analysis of the Gemmini modules was set to 100 MHz. From Table 2, as expected, the power consumption increased with an increase in the SA dimension. Doubling the SA dimension of an accelerator led to an increase in power consumption by an average factor of 3.1. For a given Gemmini SA dimension configuration, the hardware im2col module resulted in an increase in power consumption by an average factor of 1.06.

**Figure 16:**
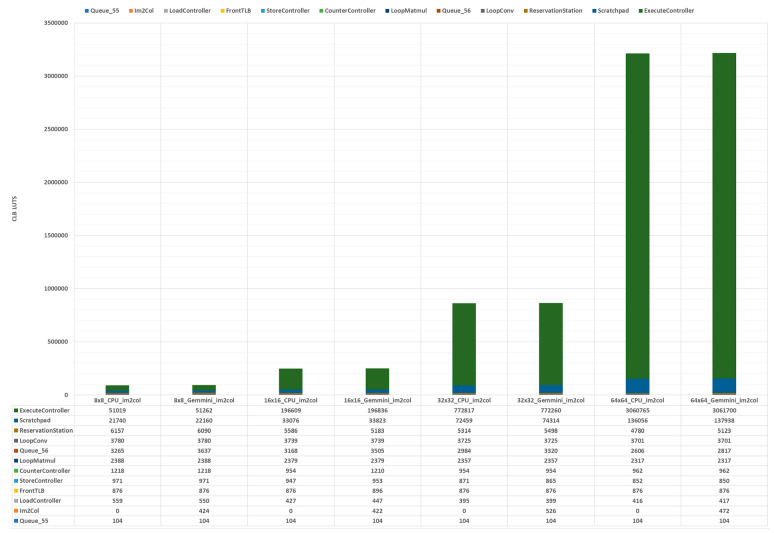
Gemmini modules CLB LUT usage.

Figure 16 illustrates the CLB LUTs consumed by each module of the Gemmini configurations detailed in Section 3. Depending on whether there existed an im2col hardware module or not, the modules were arranged from the highest consumer to the lowest consumer, as follows: ExecuteController (95.164%), Scratchpad (4.287%), ReservationStation (0.159%), LoopConv (0.115%), Queue_56 (0.086%), LoopMatmul (0.072%), CounterController (0.030%), FrontTLB (0.027%), StoreController (0.026%), Im2Col (0.015%), LoadController (0.013%), and Queue_55 (0.003%). It is not surprising that the ExecuteController and the Scratch used the most CLB LUTs because they were made up of the SA and memory elements, respectively. All the other modules used less than 0.2% of the CLB LUTs.

Overall, some important observations were made from the results of this experiment to investigate the SA dimensions and the hardware im2col module on hardware resource utilization. Some of the observations included the following: increasing the SA dimension resulted in an increase in hardware metrics, such as CLB LUTs, CLB registers, DSPs, and power, excluding the BRAM tiles and maximum frequency; for a given SA dimension, implementing the im2col module in hardware led to an increase the CLB LUTs, CLB registers, and power, while the BRAM tiles, DSPs, and maximum frequency remained unaffected; and lastly, the 64 × 64 SA dimension cannot be practically implemented in a low-cost device used for Internet of things (IoT) applications.

#### Hardware Resource Comparison

**Figure 17:**
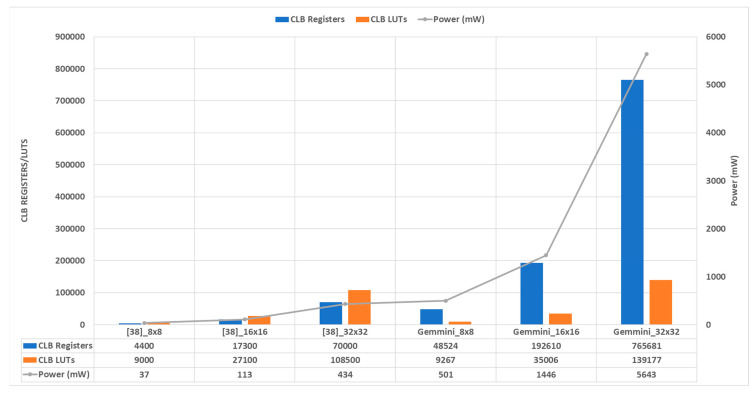
Systolic array hardware resource comparisons.

It is unfair to compare open-source DL hardware frameworks given that the design goals and implementation of the frameworks are different. The frameworks could be implemented as hardware-only, software-only, or a hardware/software co-design. The systolic array component of the generated Gemmini accelerators was compared to a DL generator proposed by Nicole et al. [40]. The work in [40] proposed a Python-based framework that is capable of generating systolic arrays for DL acceleration. The framework reduces the design and verification time by leveraging the programming abilities of the HDL embedded in Python. The framework is used to perform design space exploration of various parameters on an FPGA board. The FPGA board chosen for the work was the Zynq Ultrascale+ MPSoC ZCU104 Evaluation Board. They explored the effects of the systolic array size, PE structure, data bitwidth, and matrix sparsity on hardware metrics, such as power consumption, latency, and area occupation. For a fair comparison, the ZCU104 board was used to synthesize only the Mesh module that contains the Gemmini systolic array. Figure 17 illustrates the synthesis results of the Gemmini Mesh module and the systolic array proposed by [40].

Three systolic arrays, namely, 8 × 8, 16 × 16, and 32 × 32, each with 8-bit input data width and 24-bit output data width, were compared. The hardware resource comparison metrics were CLB registers, CLB LUTs, and dynamic power consumption. Figure 17 illustrates the hardware resources used by the systolic array of both Gemmini and [40]. From the figure, for both Gemmini and [40], the registers and LUTs increased by an average of 4× when the systolic array was doubled, while the dynamic power consumed increased by 3.4×. For the same systolic array size, Gemmini used 11×, 1.2×, and 13× the registers, LUTs, and dynamic power relative to [40], respectively.

Several reasons account for the use of more hardware resources by Gemmini as compared to [40]. The Gemmini framework focused on generating an entire SoC and for that matter, the systolic array was not optimized, while the work in [40] concentrated on only generating an optimized and efficient systolic array. Moreover, the systolic array of Gemmini is made up of other components, such as a transposer and command signal buffers. These modules help with arranging and assigning data to the systolic array of Gemmini. The arrangement and assignment of data to the systolic array in [40] are done in a testbench, and thus, are not synthesizable. These modules would increase the hardware resources of the work in [40] when synthesized together with the systolic array.

### 5.3. Gemmini Performance-Per-Area Analysis

Section 5.1 describes the performance of the GEMM operation of various matrix sizes on the Gemmini accelerators, as well as the Rocket CPU core, while Section 5.2 describes the hardware resource consumption of various Gemmini configurations. This section, therefore, describes the performance and synthesis results found when estimating a figure of merit known as the performance per area. The performance-per-area metric estimates the performance in terms of the execution time of various matrix dimensions on the generated Gemmini hardware architectures. It also estimates how each dataflow option makes use of the available hardware resources. The metric for a particular Gemmini accelerator was calculated as the ratio of the total speedup of the accelerator and the total number of CLB LUTs consumed. The speedup of a Gemmini accelerator was the ratio of the total execution time of all the GEMM operations on the accelerator and the Rocket CPU core. The speedup values are illustrated in Figure 15 of Section 5.1, while the CLB LUTs consumption is shown in Table 2 of Section 5.2.

**Figure 18:**
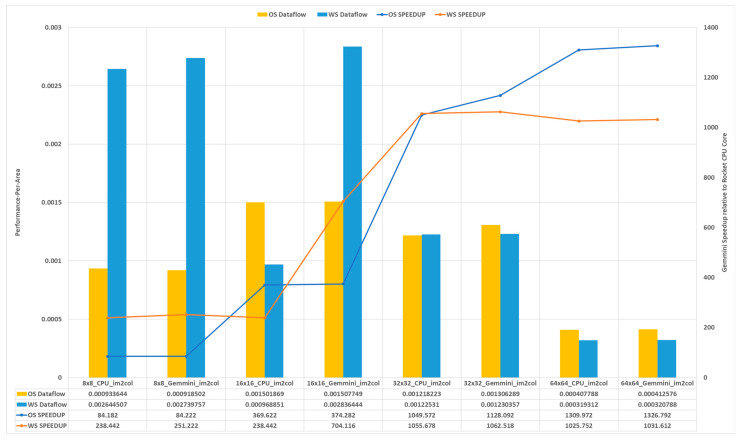
The performance per area of Gemmini configurations.

Figure 18 illustrates the performance per area of each Gemmini configuration, as well as the performance speedups relative to the Rocket CPU. From the figure, it can be seen that although the Gemmini configuration with the 64 × 64 SA dimension recorded the highest speedup, it achieved the lowest performance per area. This indicated that it recorded the best performance improvement over the Rocket CPU core by using a huge amount of hardware resources. This translated to it being the least efficient Gemmini configuration. It can also be observed that for each dataflow option, the Gemmini 16 × 16 SA dimension configuration recorded the highest performance per area, indicating that it achieved the best performance gain by not sacrificing a lot of hardware resources. The results from this experiment estimated that the 16 × 16 Gemmini SA dimension with a hardware im2col module using the WS dataflow option achieved the best performance-per-area metric among all the Gemmini configurations.

## 6. Conclusions

The Gemmini framework is a promising open-source tool for generating SAs to enable the acceleration of DL algorithms in hardware. Most of the recent works on Gemmini focus on improving individual components of the accelerator or comparing various workloads on a fixed configuration. There is, therefore, a lack of a thorough configuration space exploration of the accelerator on workloads involving the basic building block of DL algorithms, which is the GEMM operation. Moreover, the effects of different configurations of the accelerator on hardware resource consumption are not usually reported. To date, the only hardware resource utilization of the Gemmini accelerator can be found in the original paper. The paper reported ASIC results of a single Gemmini configuration ignoring FPGA results, which have become integral in prototyping DL algorithms due to their flexibility and shorter time to market. This study investigated the performance metrics of different GEMM matrix sizes on various configurations of the Gemmini accelerator. This was done to observe the effects of Gemmini configuration parameters, such as SA sizes, dataflow options, and im2col operation, on performance for various GEMM workloads. This study also aimed to serve as a milestone in mapping various Gemmini configurations on a commercial FPGA device to report their effects on hardware resources, such as area, frequency, and power consumption.

To investigate the SA dimensions (8 × 8, 16 × 16, 32 × 32, and 64 × 64), im2col module, and dataflow options (OS and WS) on performance, various GEMM sizes were run on the accelerators and the RISC-V Rocket CPU core for comparison. In terms of performance, the WS dataflow offered a speedup of 3× relative to the OS dataflow and the hardware im2col operation offered a speedup of 1.1× relative to the operation on the CPU. Some important lessons/observations were made from conducting this experiment. For the Gemmini accelerator to be effective, the GEMM sizes should be above the systolic array dimension (they are most effective when they are equal). The WS dataflow should be used for smaller GEMM sizes and the OS dataflow should be used for larger GEMM sizes. The Gemmini hardware im2col module should only be used for very large GEMM sizes. Lastly, the accelerator should not be used for GEMM dimensions below 2 × 2.

To investigate the SA dimensions (8 × 8, 16 × 16, 32 × 32, and 64 × 64) and im2col module of the Gemmini configuration options on the hardware resource utilization, the accelerators were synthesized using a Virtex UltraScale+ XCVU19P-FSVA3824-1-E FPGA device. In terms of hardware resources, an increase in the array size by a factor of 2 led to an increase in both the area and power by a factor of 3.3 and the im2col module led to an increase in area and power by factors of 1.01 and 1.06, respectively. Some important observations were made from the results of this experiment. Increasing the SA dimension resulted in an increase in hardware metrics, such as CLB LUTs, CLB registers, DSPs, and power, excluding the BRAM tiles and maximum frequency. For a given SA dimension, implementing the im2col module in the hardware led to an increase in the CLB LUTs, CLB registers, and power, while the BRAM tiles, DSPs, and maximum frequency remained unaffected. Lastly, the 64 × 64 SA dimension cannot be practically implemented in a low-cost device used for Internet of things (IoT) applications.

Using the performance and synthesis results the performance-per-area metric was calculated, which indicates how much hardware area has to be sacrificed to improve the performance of the Gemmini accelerators. The results from this experiment estimated that the 16 × 16 Gemmini SA dimension with a hardware im2col module using the WS dataflow option achieved the best performance-per-area metric among all the Gemmini configurations. This indicated that it achieved the best performance gain by not sacrificing a lot of hardware resources.

## Figures and Tables

**Table 1:** GEMM workload performance on Gemmini and the Rocket CPU core.

Spatial Array Dimension	Matrix Dimension	Execution Times (Cycles)				
		Gemmini Accelerators				Rocket CPU
		OS Dataflow		WS Dataflow		
		im2col in CPU	im2col in Hardware	im2col in CPU	im2col in Hardware	
**8 × 8**	**2 × 2**	809	809	329	329	167
	8 × 8	777	777	388	388	7227
	16 × 16	1341	1341	500	497	53,662
	32 × 32	3491	3491	1242	1173	410,974
	128 × 128	104,957	104,863	36,228	34,196	26,624,924
16 × 16	2 × 2	815	815	358	358	171
	8 × 8	785	785	401	401	7252
	16 × 16	879	879	475	475	53,671
	32 × 32	1477	1477	732	712	411,120
	128 × 128	17,761	17,489	11,172	9471	26,628,902
32 × 32	2 × 2	891	891	482	482	140
	8 × 8	901	901	472	472	7220
	16 × 16	942	942	560	560	53,672
	32 × 32	1116	1116	706	706	410,955
	128 × 128	5531	5114	5808	5765	26,628,857
64 × 64	2 × 2	1002	1002	604	604	157
	8 × 8	1021	1021	619	619	6844
	16 × 16	1068	1068	686	686	49,375
	32 × 32	1248	1248	969	969	379,797
	128 × 128	3955	3902	5263	5230	24,491,236

**Table 2:** Gemmini hardware resource consumption.

Gemmini Configuration	CLB LUTs (Utilization)	CLB Registers (Utilization)	BRAM Tile (Utilization)	DSPs (Utilization)	Frequency (MHz)	Power (W)
8 × 8_CPU_im2col	90,165 (2.21%)	34,298 (0.42%)	96 (4.44%)	149 (3.88%)	52.6	1.010
8 × 8_Gemmini_im2col	91,695 (2.24%)	34,414 (0.42%)	96 (4.44%)	149 (3.88%)	52.6	1.064
16 × 16_CPU_im2col	246,108 (6.02%)	65,545 (0.80%)	128 (5.93%)	171 (4.45%)	47.6	2.073
16 × 16_Gemmini_im2col	248,239 (6.08%)	65,237 (0.80%)	128 (5.93%)	171 (4.45%)	47.6	2.141
32 × 32_CPU_im2col	861,560 (21.09%)	180,311 (2.21%)	64 (2.96%)	204 (5.31%)	47.6	6.963
32 × 32_Gemmini_im2col	863,585 (21.14%)	179,171 (2.20%)	64 (2.96%)	204 (5.31%)	47.6	6.581
64 × 64_CPU_im2col	3,212,384 (78.62%)	602,962 (7.38%)	128 (5.93%)	267 (6.95%)	55.6	25.854
64 × 64_Gemmini_im2col	3,215,870 (78.71%)	600,044 (7.34%)	128 (5.93%)	267 (6.95%)	55.6	31.240

## Data Availability

Not applicable.
